# Editorial Correspondence

**Published:** 1857-02

**Authors:** W. F. Westmoreland

**Affiliations:** Paris


### EDITORIAL AND MISCELLANEOUS.

*Editorial Correspondence.*

PARIS, Nov. 15, 1856.

*Dear Doctor*—The School of Medicine was officially opened to-day. The exercises, or, perhaps I should rather say, the official robing of the Faculty, and numerous *agregés*, was very imposing.

The form differed but little from that usually observed upon such occasions in America. The introductory was delivered by M. Natális Guillot; and, from the subject of the address, was received with considerable favor, it being an Eulogy upon the Character and Attainments of M. Requin, who died in 1854, and whose Chair he now fills.

From a recent decree of the Faculty, many were disappointed in obtaining admittance to the hall. It appears, that two years ago, during the introductory address, there was such a disturbance by the students, as to prevent the possibility of its delivery; since which time, to obtain admittance to the introductory exercises, it is necessary to present a card from the Secretary, or a note of invitation from the Dean, with the bearer's name. Learning the above regulation at an early hour, this morning, I was fortunate enough to obtain a note of invitation, from M. Dubois, Dean of the Faculty.

It was said yesterday and this morning, that M. Guillot would likely be disturbed during his introductory, which, however, from the subject of the address, or other considerations, did not occur; not that he is personally unpopular, but, that the manner in which he received his appointment to the Faculty, renders his position unpopular. Heretofore when a Chair became vacant, the applicants, and there were usually quite a number, were submitted to the most rigid test of qualification, and the one thought best qualified by the committee appointed to conduct the *concour*, received the position. At present the *concours* for members of the Faculty, have been suppressed—the Emperor reserving the right to fill the vacancies. M. Guillot received his appointment from the Emperor.

The Latin Quater, in which is located the public Institutions, both Law and Medical, has, for the past few days, presented rather a lively appearance. The students are returning in numbers, from their homes in the provinces, or other points, where they have spent their vacations; their gay re-unions, lively songs, &c., &c., has, as above suggested, changed to some extent, the aspect of this monotonous Quater.

The various Clinical Professors, for the past ten days, have been gradually assuming their respective duties; and after to-day, all will commence their regular course of instruction. So, you see, that the winter course is, at last, regularly under-way. Not that the two months' vacation has been lost to those, who were disposed to work, as the same materials have existed; the hospitals, all the time open, and under the control of young talented physicians and surgeons, who are constantly laboring for character and position.

The city of Paris for the past month, has been remarkably healthy. With the exception of an epidemic of Puerperal Fever, at the Maternite and a few lying in beds at Hôtel Dieu, there has not been the least tendency to an epidemic of any kind, in several months. As an evidence of the healthy condition of the city, it is only necessary to say, that at La Charêté, more than once, within the past few weeks, three and four days have passed, without a death—something rare in a hospital of five or six hundred beds.

The epidemic of puerperal fever above alluded to, was of a very malignant character; its ravages being so great that the Maternite was closed. It was in the lying-in ward of M. Trousseau, at Hôtel Dieu, which was afterwards closed, that I saw this fatal disease. Almost every case proved fatal in from two to four days; it is true, that some who complained of more or less pain, and had slight fever, recovered; but all who had well marked symptoms of puerperal intoxication, if you will allow the expression, died. The post-mortem examinations revealed quite a variety of lesions, all, however, traceable to one general source. In quite the majority of cases, inflamma tion of the uterus and peritoneum was well marked; but in a number of cases, not sufficiently extensive to account for the symptoms and death of the patient—having the appearance of being rather an extension than the original seat of the inflammation. Abscesses in the various tissues, frequently in the joints, and readily detected before death, were not unfrequent; dependent, without doubt, upon a purulent intoxication, as pus was found in the veins of the uterus.

The plan pursued by M. Trousseau, whether objectional or not, will likely be objected to by those who regard every case of puerperal fever, as a symptomatic fever, dependent upon an acute inflammation of the uterus or peritoneum, and, for the arrest of which, active antiphlogistic remedies must be brought to bear. Small doses of calomel, frequently repeated; tonics, such as quinine, &c.; frictions frequently repeated over the abdomen, with opium and belladonna, followed by poultices, constituted in a number of cases, the treatment. To a question asked, if he ever bled in such cases, he remarked, that he never did—that the patient would then have to recover from the loss of blood as well as the disease.

On the 10th inst., I visited the wards of M. Becquerel, at La Pitié. Since the death of that great and good man, M. Valleix, M. Becquerel may be considered as one of the most practical, as well as one of the most popular lecturers upon internal pathology, in Paris. I found his wards crowded, and amphitheatre crowded, with medical students, the latter to such an extent, that many were disappointed in obtaining seats. His lecture, which was the first of the present course, was introductory to a series of lectures upon *the diseases of the uterus.* The field laid out was sufficiently extensive—embracing all the lesions of this important organ. The obscurity of some of the diseases of the uterus; the diversity of opinions in regard to the treatment of others better understood, as well as the extreme frequency of such affections, renders this a very important course—one that I should take great pleasure in following, were it possible. I am happy, however, to be able to announce, that I have made an arrangement with Dr. Wm. Hart of New Orleans, to report the entire course of lectures, for the Atlanta Medical & Surgical Journal. Dr. Hart has been in Paris near five years, and from his intimate knowledge of the French language, as well as the science of medicine, will be able to give a thorough exposition of the views of M. Becquerel. I look upon the above arrangement as quite an addition to our Journal for 1857. The reports will commence with the April or May number.

In passing through the wards of M. Piorry, a few days ago, I was attracted by a slight operation which was being performed, the result of which illustrates, in a very striking manner, the danger of opening, by incision, abscesses of the liver —a danger not generally well understood by young practitioners; and, by quite a number more experienced, if understood, not regarded. The case in point, was an abscess of the liver; the precise character of which, from the rather obscure history, could not be determined. It was to render more complete the diagnosis, that the operation was performed, which consisted in a simple puncture of the abscess with a small exploring trocar. After the discharge of a small quantity of thick, healthy pus, the result evidently of an inflammation of the organ, the canula was withdrawn. In a few hours the patient was attacked with violent peritonitis, of which, he died in two or three days. The principle that I here wish to impress, was discussed before the operation, but it was imagined, that the puncture made in the walls of the abscess proper, by the small trocar, would not be sufficiently large for the escape of pus, the result, however, proved that a sufficient quantity escaped to set up an inflammation, which, proved fatal in a very short time.

I know physicians who pretend to science, and in a general way are successful practioners, who never, for a moment, stop to determine whether there be adhesions between the peritoneum covering the walls of the abdomen, and that covering the liver, but alike in all cases where there is a prominence and fluctuation, make full use of the bistoury. In all cases, if there be much distension, the incision is followed by a free flow of pus, which satisfies both physician and patient for the time, but if there be no adhesions, as soon as the abscess is to a certain extent emptied, the parallelism between the opening in the walls of the abcess and that in the walls of the abdomen is lost, and the remainder of its contents is discharged into the peritoneal cavity. The result, under such circumstances, is sufficiently well illustrated in the above case. It is always best, if it is desirable to reach the collection, to do so by a slower but safer process—by means of caustics, thus insuring adhesions before reaching the contents of the tumor.

The discussion at the *Adcadémie de Médecine*, upon ovarian cysts, is still continued with the same interest. It would give me great pleasure to keep you informed in regard to the discussion, but to give you any thing like a correct idea of the positions and variety of opinions of the various disputants, would require more time and space than I can possibly devote to any one subject. At the close of the discussion, I may give a short resúme of the more important points discussed.

Speaking of ovarian cysts, reminds me of two cases of this, at present, interesting affection that I have observed since the commencement of the discussion, and which, from the unhappy termination of one, and the critical position of the other, are not without interest. One was in the wards of M. Piorry, the other under the care of M. Velpeau. The former was a cyst of very great size, and had been tapped twenty-five times without the least accident following any one of the punctures. M. Piorry proposed to draw off the water, and inject the sac with iodine. The cyst was emptied by means of an ordinary trocar, but the injection of iodine, from some defect about the syringe was deferred. Within the first twenty-four hours after the puncture, there was set up, a violent inflammation of the sac and peritoneum, which proved fatal in three or four days. Had the iodine been injected, the result would, in all probability, have been the same; and then, all would have readily agreed that the inflammation and death, was the result of the injection.

The case, in the wards of M. Velpeau, presented a much smaller cyst than the one above alluded to. It appears that some four or five weeks before my attention was called to this case, which was on the day of the second operation, a first puncture was made, and iodine injected without the least unpleasant symptom. The second puncture, as the first, was made with a common sized trocar; after the discharge of several pints of limpid serum, he injected a pint of tepid water to wash out the sac, that the iodine might more readily act. From the extreme difficulty in extracting the water injected, and for which M. Velpeau could not account, the injections of iodine was deferred. Acute inflammation of the sac and peritoneum followed, and at present, ten days after the operation, the patient is in an extremely critical position. The same may be said of this case, as the one under the care of M. Piorry; and from the two, it would appear that it is more than probable that many of the accidents attributed to the injection of iodine, would have taken place without its use.

---

PARIS, Nov. 25th, 1856.

As the remarks that I may make, from time to time, will have, to a very great extent for their basis, the clinics of M. M. Nelaton and Velpeau, it will perhaps not be amiss here, for me to say something of these two distinguished Surgeons; not as to whether they have blue or black eyes, handsome or ugly faces, but something more important—something by which one may be able to judge of the importance that should be attached to their peculiar views or doctrines.

There are, as you are apprised, several classes of surgeons: one class, for example, who believe strongly in the bistoury, and never let a chance slip without using it, if they can by any process of reasoning, however far-fetched, make it the least apparent that an operation is justifiable. This class of surgeons are always looking out for difficult and terrible operations, and if they have but seldom or never been performed, they are so much the better pleased. There is another class at just the opposite extreme, who, perhaps, are as irrational and as apt to lead astray as the former.

It is evident that, upon quite a number of subjects, the opinions of the above two classes of surgeons, would be widely different; and that our deductions, from a proposition emanating from a member of either, would depend greatly upon the class to which he belonged.

Between these two extremes, we find the rational and true surgeons; and of these, at Paris at least, we may make, with propriety, two classes: One who hold on with considerable tenacity, to the doctrines and precepts of the old school, contending that it is better to follow an old and well-beaten track, if it is a little longer and more tedious, than to attempt a shorter and more expeditious route, and run the risk of being ship-wrecked on the way. They, however, after the track has been surveyed, and found practicable, follow with considerable grace. They may be denominated the conservative surgeons. The other class is less conservative—one not willing to receive the doctrines of the old school without investigation—one disposed to make progress—to experiment where it can be done without any great risk to the patient. At the head of the former, we find M. Velpeau ; and with the latter, but perhaps more conservative than the majority of this class, we find M. Nelaton. It is unnecessary for me to speak of the attainments of M. Velpeau, as he is known by all. He has certainly done much for science, and enjoys at present, and deservedly so, the most enviable reputation of any surgeon living, it being as extensive as civilization itself. It is true that he is strictly conservative, but in this way, I am sure that he has done much for science, by acting as a stay to those of the young school, who would go too fast—arriving at conclusions without sufficient data. M. Nelaton is a link uniting the old and new schools, combining the good qualities of both, with but few of their faults. I consider him one of the most practical surgeons in Europe. He is a most excellent teacher, and fine operator—two qualities important in a clinical professor ; and which renders his clinics exceedingly popular, his wards and amphitheatre being all the time crowded with students, as well as the profession of the city. But I must stop, as I have already extended these remarks much longer than I anticipated, for which I ask pardon.

There is, at present, in the wards of M. Nelaton, one or two cases of considerable interest. The first that I shall notice, is a very common affection, but from the treatment proposed, is certainly not without interest. It is a case of hereditary syphilis in a girl twelve or thirteen years of age, which has resisted all the usual plans of treatment. Caries of the bones of the cranium, and of the nasal bones, nodes and gummy tumors, &c., at various points, with the fact, that some years ago, the disease, once or twice, yielded to anti-syphilitic remedies, leaves no doubt as to the character of the affection. She has now been in the hospital several months, and notwithstanding the most active and appropriate anti-syphilitic remedies have been constantly administered, the disease has steadily progressed. M. Nelaton proposes in this case, syphilisation as it is called, which is, as you are apprised, a sort of saturation of the system by means of numerous inoculations with the virus of a primitive chancre. The original proposition was to render the system refractory to syphilis, by repeated inoculations of the syphilitic virus; when they arrived at the point where the inoculations would no longer take effect, the subject was said to be in a state of syphilisation. The proposition was first made by M. Anzias Turenne, of Paris, about the year 1850, if I am not mistaken, as a prophylactic, as vaccination in small pox. Soon after, M. Sperino, of Turin, M. Morchal, (de Calvi,) of Paris, and some others applied it to the treatment of syphilis, and as they contended, with some results. In this state, in 1852, without any fixed or general laws, with but few friends or data, it went before the *Académie de Médecine*, and there received, as it was thought by all, its death-blow. The contempt with which it was received, and the anathemas hurled against it by that savant body, paralyzed even the warmest supporters of this new doctrine; so that in France, since that decision, nothing has been heard of syphilisation, at least publicly, until M. Nelaton's lecture, a few days ago, upon the case now under consideration. It appears, however, that the physicians of other countries have not been idle; that experiments, to test the value of the proposition, have been constantly going on.

M. Bœck of Christiana, has, perhaps, experimented more extensively than any other physician. He has recently published a brochure, of forty or fifty pages, in which is contained in detail, a number of experiments upon children. It was the result of these experiments that called M. Nelaton's attention to this subject; and if they are to be relied upon, and from the position of M. Bœck, we cannot well doubt them, there will, yet, something practical grow out of this, at first, apparently absurd idea. But to the case under consideration: The little girl has now been under treatment fourteen or fifteen days; from three to five inoculations have been made every two or three days; at first with the virus of a primitive chancre, more recently with the pus from the first inoculations. The inoculations are principally upon the arms and trunk. She has, at present, several crops of beautiful chancres. About the time of the appearance of the chancres, from the second inoculation, there was some constitutional disturbance; more or less fever, for twenty-four or thirty-six hours, which was regarded by M. Anzais-Turenne, who is conducting the experiments, as a favorable indication. The patient is, at present, as cheerful as usual. I shall keep you informed of the progress of this case, from which so much is expected. It is, perhaps, not amiss here, to state, that according to the views of M. Bœck, this case, owing to previous treatment, which I am told has been to some extent, mercurial, is not a very favorable one to test the value of this new treatment—the results not being so satisfactory in cases where mercury has been previously administered.

A resúme of the pamphlet above alluded to, was published a few days ago, in the *Archives Générales de Médecine*, a translation of which, by J. J. West, M. D., of Savannah, I send you by to-day's mail.

The other case above alluded to, in the wards of M. Nelaton, is also not an unfrequent lesion; but in connection with the precepts of this distinguished surgeon, will, I hope, not be without interest. It is a traumatic aneurism of the bronchial artery, the result of an unfortunate venesection; the aneurismal tumor had been mistaken for an abscess, and an incision made, followed by a considerable hemorrhage, which was, however, readily arrested by compression over the point incised. Upon entering the hospital, which was some days after the incision, there was found, at the head of the above tumor, something, larger than a hen's egg, presenting all the signs of an aneurism: at the most prominent point, an incision from half an inch to an inch in length, had been practiced, through which could be readily seen the clot by which the hemorrhage was arrested.

What should be the practice in such cases? Shall we ligate the artery by the *ancient* or by the *modern* method, as they are called by the classics; but more properly, I think, by M. Vidal, denominated the *direct* and the *indirect* method? That is, shall we cut down upon the wounded vessel, opening freely the aneurismal sac, and ligate both ends of the artery, or shall we adopt the *modern* method—the *indirect* of M. Vidal—ligating the artery above the tumor? M. Nelaton said, that in a parallel case, he had once adopted the latter method, and in a few days had a secondary hemorrhage; he ligated the vessel a second time nearer the trunk, and a few days after, had the same accident—the hemorrhage being from the distal extremity of the wounded vessel, making it necessary, at last, to open the aneurismal sac, and ligate both extremities of the artery. In the case under consideration, he made a free incision through the tumor, turned out the clot, and ligated both ends of the artery. The eleventh day after the operation, the ligatures were detached without any thing unpleasant; and now, the twentieth day after the operation, the wound has almost entirely healed. M. Nelaton contends, that the danger so much dreaded by some surgeons, of a softening and rupture of the artery, ligated when bathed in pus, is all imaginary. He says, that he has never yet met with such an accident where the artery was previously healthy, and he has ligated many surrounded by extensive abscesses. It is unnecessary to say, that in the above case, but for the incision in the aneurismal sac, the treatment would have been different.

Yours, &c.,

W. F. WESTMORELAND.

